# On the Soil Roughness Parameterization Problem in Soil Moisture Retrieval of Bare Surfaces from Synthetic Aperture Radar

**DOI:** 10.3390/s8074213

**Published:** 2008-07-15

**Authors:** Niko E.C Verhoest, Hans Lievens, Wolfgang Wagner, Jesús Álvarez-Mozos, M. Susan Moran, Francesco Mattia

**Affiliations:** 1 Laboratory of Hydrology and Water Management, Ghent University, Coupure links 653, B-9000 Ghent, Belgium; E-mail: Hans.Lievens@UGent.be (H.L.); 2 Christian Doppler Laboratory, Institute of Photogrammetry and Remote Sensing, Vienna University of Technology (TU Wien), Gusshausstraβe 27-29, 1040 Vienna, Austria; E-mail: ww@ipf.tuwien.ac.at (W.W.); 3 Department of Projects and Rural Engineering, Public University of Navarre, Los Tejos, Arrosadia s/n, 31006 Pamplona, Spain; E-mail: Jesus.Alvarez@unavarra.es (J. A.-M.); 4 USDA ARS Southwest Watershed Research Center, 2000 E. Allen Rd., Tucson AZ 85719, Arizona, USA; E-mail: Susan.Moran@ars.usda.gov (S.M.M.); 5 Consiglo Nazionale delle Ricerche (CNR), Istituto di Studi sui Sistemi Intelligenti per l'Automazione (ISSIA), via Amendola 122/D, I-70126 Bari, Italy; E-mail: Mattia@ba.issia.cnr.it (F.M.)

**Keywords:** Soil roughness, Soil moisture retrieval, Synthetic Aperture Radar, Uncertainty

## Abstract

Synthetic Aperture Radar has shown its large potential for retrieving soil moisture maps at regional scales. However, since the backscattered signal is determined by several surface characteristics, the retrieval of soil moisture is an ill-posed problem when using single configuration imagery. Unless accurate surface roughness parameter values are available, retrieving soil moisture from radar backscatter usually provides inaccurate estimates. The characterization of soil roughness is not fully understood, and a large range of roughness parameter values can be obtained for the same surface when different measurement methodologies are used. In this paper, a literature review is made that summarizes the problems encountered when parameterizing soil roughness as well as the reported impact of the errors made on the retrieved soil moisture. A number of suggestions were made for resolving issues in roughness parameterization and studying the impact of these roughness problems on the soil moisture retrieval accuracy and scale.

## 1. Introduction

Although surface soil moisture only constitutes 0.0012% of all water available on Earth [1], it plays an extremely important role in different hydrological processes. During precipitation events, soil moisture controls the infiltration rate, and consequently the amount of runoff produced. The latter process greatly influences erosion processes and determines resulting flood events. The wetness of the soil also controls the evapotranspiration rate and thus the micro-meteorology. Especially information on the spatial distribution of soil moisture, caused by micro-topography, vegetation, and stochastic precipitation events, is of major importance for watershed management, as it allows for optimizing the reallocation of water supplies during dry periods, or aids in predicting and managing high tides and floods during extreme rainfall events. From an agronomic point of view, soil moisture is a crucial variable for crop development and is used to monitor crop temporal and spatial variation for important management decisions related to irrigation scheduling and precision farming.

Remote sensing offers the potential for monitoring surface features at the regional scale. Particularly, sensing in the microwave region may deduce spatial soil moisture information as the detected microwave signal is in part influenced by the dielectric properties of the soil, and thus the moisture content [2-4]. For operational purposes, spaceborne platforms are preferred as they allow for a global coverage at regular time intervals [5]. The only satellites that can currently meet the spatial resolution requirements needed for capturing small-scale soil moisture patterns are active microwave sensors, of which Synthetic Aperture Radar (SAR) is the most common imaging active microwave configuration [6]. However, the temporal coverage needed for many hydrological modeling applications (1-5 days repeat cycle [7]) require temporal coverages which currently cannot be met by most available SAR sensors.

Due to the coherent measurement process of SAR instruments, the superposition of waves reflected by scatterers at the earth's surfaces leads to a grain-like appearance of SAR images (“speckle”) and a high sensitivity of the measurements to the geometric arrangement of the scatterers [8,9]. As a result, SAR measurements are very sensitive to soil roughness, which in agricultural fields is affected by the characteristics of tillage [10-21]. Consequently, the parameterization of surface roughness and its spatial variability can pose major problems for soil moisture retrieval [5,19,22]. As such, accurate soil moisture retrieval with single-frequency, single incidence angle, single-pass SAR imagery is not possible without *a priori* soil roughness information [6]. Furthermore, if the soil is vegetated, additional information is needed with respect to the vegetation parameters (such as fresh biomass, canopy structure, …) in order to retrieve soil moisture.

The backscattered signal from a bare soil depends on a combination of factors, including radar properties (frequency, polarization), surface characteristics (dielectric constant of the soil, and by consequence soil moisture, and surface roughness), and the incidence angle of the incoming microwave [3,15]. Different models have been proposed that relate the dielectric constant to the soil moisture content. For soil moisture retrieval studies, the following models are mainly used: the polynomial expressions fitted by Hallikainen *et al.* [23] and the semi-empirical four-component mixing model developed by Dobson *et al.* [24]. The latter model, valid for frequencies larger than 4 GHz and smaller than 18 GHz, was further extended for the 0.3 to 1.3 GHz range by Peplinsky *et al.* [25,26].

With respect to soil moisture retrieval, one of the first studies, carried out by Ulaby and Batlivala [27] found that the optimal radar configuration consists of a co-polarized (HH or VV) sensor operating at C-band at a 7° to 15° incidence angle. For this configuration, the sensitivity of the backscattering coefficient to soil roughness is minimized. At higher incidence angles, the radar return was found to be much more sensitive to surface roughness [12,28-30]. For cross-polarizations, some studies suggested a larger sensitivity to soil moisture [31] and reduced roughness effects [32,33], however, the results of these studies were inconsistent [34]. According to Holah *et al.* [30], the HH and HV polarizations are more sensitive to soil roughness than the VV polarization. These findings with respect to the co polarizations were not confirmed by Baghdadi *et al.* [35] when studying an assembled database of ERS-2, RADARSAT-1 and ENVISAT data. They discovered that the sensitivity of the radar signal to soil moisture was not very dependent on polarization.

Soil moisture retrieval from sensors characterized by a shorter wavelength than C-band is hydrologically less interesting due to the small penetration depth of the microwaves [3]. At spectral bands with a longer wavelength, more soil profile information is contained in the backscattered signal [3]; however, the sensitivity to roughness becomes more important than in C-band [36], requiring more accurate roughness information for retrieval studies to obtain accurate soil moisture values.

For very wet soils, having moisture contents larger than 35 vol%, the radar signal becomes less sensitive to soil moisture [30,37-39]. Consequently, it is difficult to accurately map higher soil moisture contents [35]. Furthermore, since under these circumstances, the penetration depth of the incident wave is minimized, the retrieved soil moisture becomes hydrologically less interesting because the information only refers to a very thin layer. The hydrological relevancy is furthermore weakened due to the aforementioned uncertainty on the retrieved value caused by the low sensitivity of the radar backscattering coefficient to high soil moisture contents.

As stated before, the parameterization of roughness is an important aspect in retrieval studies. Generally, roughness is described as a zero-mean Gaussian random process, characterized by the root mean square (RMS) height, *s*, the correlation length, *l*, and an autocorrelation function (ACF) of the surface height (e.g. [3,40], amongst many others). The latter is generally an isotropic predefined function (exponential or Gaussian), such that surface roughness characterization requires the parameterization of *s* and *l*. It is obvious that both values need to be known in order to invert the backscattering coefficient to soil moisture, *Mv*. If not, a combination of different frequencies, polarizations or incidence angles is necessary to solve for *Mv*, *s*, and *l* simultaneously [35,41-47]. However, such configuration is yet not applicable to most satellite systems [5,35], and therefore, soil roughness parameterization remains an essential part of the retrieval process. For current spaceborne systems, Bryant *et al.* [48] reported that the main source of retrieval errors were due to differences in soil roughness parameters resulting from different measurement techniques and roughness transect analyses. The discrepancies found are mainly related to the uncertainty in the measured roughness parameters, especially with respect to the correlation length [49,50].

Accurate retrieval of soil moisture is hampered not only by roughness parameterization, but also by the assumption of ideal soil characteristics by most surface scattering models which is often not fulfilled for real circumstances [21]. For instance, the assumption that soil roughness is well described by a single-scale stationary process, fails to accurately account for the complex geometry of natural soil surfaces [5]. For agricultural surfaces, roughness is often anisotropic and can be approximated by the superposition of a single-scale process related to the tillage state with a multi-scale random fractal process related to field topography [19,51]. Although the introduction of multi-scale roughness descriptions brought a more realistic representation of soil roughness, beneficial for improving the understanding of backscattering mechanisms over bare soils [22,52,53], these approaches usually involve a larger number of roughness parameters, which cause difficulties for inverting the radar signal to soil moisture [52,54]. Fortunately, the choice between a single-scale or multi-scale roughness description is not critical for inversion studies, in the sense that an equivalent description of surface scattering in terms of *s* and *l* parameters can always be sought [55,56].

Although several acceptable retrieval results have been published (e.g. [18,56-60]), numerous publications reported on poor results when inverting the backscattering coefficient to soil moisture (e.g. [17,36,61-65]). Generally, an unsuccessful roughness parameterization was assigned as being the main source of error that caused the failure of the retrieval. In order to circumvent these problems, different strategies have been suggested, consisting of calibrating soil roughness parameters (e.g. [66]), the introduction of semi-empirical relations between correlation length and RMS height [62,63], or change detection analysis under the assumption of constant roughness [67,68].

In this review, we focus on the impact of soil roughness measurement errors on the retrieval of soil moisture. A literature review on roughness characterization methodologies and corresponding problems is performed, and the impact of this parameterization on soil moisture retrieval from single configuration (1 frequency, 1 polarization) SAR imagery is assessed. The remainder of this paper is structured as follows. Section 2 briefly reviews the models used for soil moisture retrieval, with special attention to soil roughness. Section 3 discusses the parameterization of soil roughness, whereas section 4 elaborates on the impact of different soil roughness parameterization methodologies and the effect of spatial and temporal roughness characteristics on the retrieval of soil moisture. In section 5, a review of alternative soil roughness characterization methodologies or techniques for circumventing this characterization is given. Finally, conclusions are formulated.

## 2. Soil moisture retrieval

A wide range of models, ranging from experimental relationships to physically-based models have been developed in order to invert the backscatter coefficient to soil moisture. In the following subsections, a brief overview of these modeling efforts with a special focus on the roughness assumptions is made.

### 2.1. Empirical models

**Figure 1.**
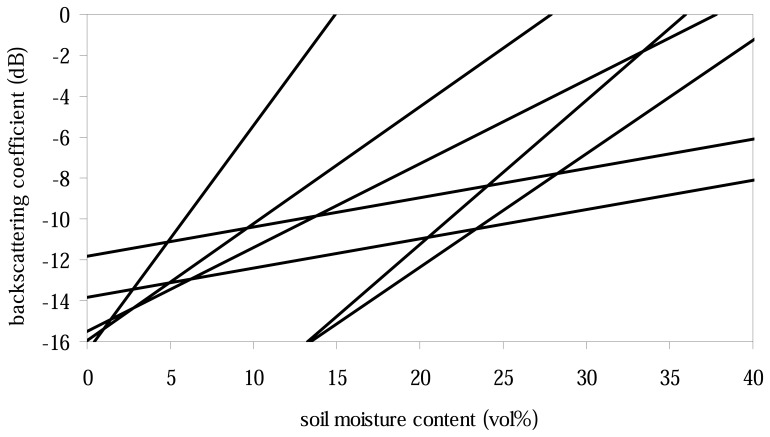
Linear relationships reported by [73,83-86] between topsoil volumetric moisture content of (nearly) bare soil surfaces and the ERS-1 backscattering coefficient.

Empirical relationships between the radar backscattering coefficient and soil moisture have been presented by several authors (e.g. [14,52,69-77]). For a bare soil, there exists a functional relationship between the topsoil moisture content and the backscatter coefficient, which also includes a surface roughness term [78]. Under these conditions, many studies have shown that a linear relationship between the backscattering coefficient and soil moisture content is a reliable approximation for one study site, if roughness is assumed to remain constant between successive radar measurements [75]. The coefficients of this linear relation have been observed to vary strongly for different study sites. This is illustrated by figure 1 which draws a number of relationships reported for ERS-1. Therefore, it is generally not possible to apply relationships observed over one area for radar backscatter inversion in other areas [14,24,74,79-81]. For other situations, new relationships need to be established through calibration work [35,60,75] or through estimating the linear model parameters from the SAR observations themselves. The latter approach has been found to work well if several dozens of SAR images are available over the study area [82]. Unfortunately, such long SAR time series are generally not available because most spaceborne SAR systems are designed to achieve a high spatial resolution at the expense of swath width and recording capacity [82].

In order to make empirical relationships more widely applicable, an exponential term can be added to the linear relationship that describes the roughness influences on the backscattering coefficient [46,52,60], leading to:

(1) $$\sigma^{0}\;(\text{dB})=a+b\;Mv+ce^{‐ks}$$

with *a*, *b*, and *c* calibration coefficients, and *k* the wave number (*k* = 2π/λ, λ being the wavelength). Yet, such models still receive limited use, since for a given radar wavelength, the calibration coefficients remain dependent on the polarization and incidence angle [30,47,60,64]. Furthermore, this relationship does not account for the spatial properties of soil roughness, as described by the correlation length and autocorrelation function.

### 2.2. Semi-empirical models

Many semi-empirical models based on a theoretical foundation and model parameters derived from experimental data have been developed [6], yet only a few are commonly used. The most popular models are those developed by Oh *et al.* [14] and Dubois *et al.* [80,87].

The Oh model uses ratios of measured backscattering coefficients in different polarizations. Because the large database of polarimetric data (L-, C-, and X-band) from truck-mounted scatterometer experiments, used to parameterize the model of Oh *et al.* [14], consisted of soil surfaces with approximately the same correlation length, the Oh model should be restricted in its application to surfaces of the same type as the ones used in the experiment [88], being 0.1 < *ks* < 6.0, 2.6 < *kl* < 19.7, 0.09 < *Mv* < 0.31 and 10° < θ < 70° [14], with *k* the wave number and θ the incidence angle. The Oh *et al.* [14] model was further improved to incorporate effects of incidence angle [14,89], and to model cross-polarized backscatter coefficients [90]. Oh [91] ultimately introduced a new formulation in the model such that the correlation length could be ignored.

Applying Oh's model to SIR-C measurements over the Little Washita River watershed, Wang *et al.* [71] found that the modeled cross-polarized (*q* = σ^0^_HV_/σ^0^_VV_) and co-polarized (*p* = σ^0^_HH_/σ^0^_VV_) ratios did not correspond to the ones obtained by the radar imagery. When the algorithm was applied to the entire test site, only a small percentage of pixels resulted in a normal solution. A similar problem was reported by Ji *et al.* [92], and was related to larger uncertainty of the cross-polarized compared to the co-polarized backscatter because of the poorer signal-to-noise ratio. Using airborne C-band SAR data, van Oevelen and Hoekman [93] obtained soil moisture estimates that in general were too high. Boisvert *et al.* [57] found that Oh's model systematically underestimated the backscattering coefficient for Ku-, C- and L-band data, applied to incidence angles ranging between 15° and 30°. Baghdadi and Zribi [94] found a systematic overestimation of the cross-polarization ratio which was independent on the RMS height, soil moisture or incidence angle, whereas the co-polarized ratio was correctly simulated for C-band SAR. Álvarez-Mozos *et al.* [95] reported an underestimation of the backscattering coefficient at HH polarization and C-band for smooth surfaces observed at low incidence angles, whereas the model yielded adequate results for rough surfaces and large incidence angles. D'Urso and Minacapilli [96] carried out a calibration procedure in order to use Oh's model to retrieve soil moisture values without *a priori* knowledge of roughness parameters. They used a SIR-C/X-SAR scene and calibrated the model for two agricultural fields where soil moisture data were available. Results were compared to moisture estimates obtained from a hydrological model, yielding better results for L-band data than for C-band. The results obtained were strongly influenced by the vegetated cover of the fields. Fung and Chen [97] reported that Oh's model fitted observations well over large angles of incidence, but less well over small incidence angles, especially for low frequency data. They observed that the largest errors were obtained at incidence angles of about 10°. At higher frequencies, the model performed better.

The Dubois model relates the HH or VV polarization to the soil's dielectric constant, surface roughness, incidence angle and radar frequency. Basically, for a given radar configuration and soil roughness, this model linearly relates the dielectric constant of a soil to the backscattering coefficient, expressed in dB. Dubois *et al.* [80] restricted the validity of the model to *ks* < 2.5 and incidence angles larger than 30°.

Ji *et al.* [92] applied Dubois' model and discovered a high error in the retrieved soil moisture for C-as well as L-band imagery. Wang *et al.* [71] applied the model to SIR-C images over the Little Washita River catchment and found that the model allowed for soil moisture inversions with an accuracy of 5.9%. However, the authors also mentioned that the inversion procedure did not bear a solution for a substantial number of bare soil pixels. Baghdadi and Zribi [94] generally found an overestimation of the radar backscattering coefficient of 1.7 dB in HH polarization and no bias for VV polarization. Their simulations showed an underestimation of the backscattering coefficient for smooth surfaces (*s* < 0.6 cm), an overestimation for surfaces with an RMS height larger than 1.6 cm, and a correct simulation for surfaces with an intermediate roughness. For soil moisture contents smaller than 30%, Baghdadi and Zribi [94] observed an overestimation of the backscattering coefficient. For higher moisture contents, no bias was found. Álvarez-Mozos *et al.* [95] applied Dubois' model and obtained poor results for low incidence angle RADARSAT-1 data. A theoretical analysis showed a better performance for large incidence angles although the authors reported an unrealistic sensitivity of the backscattering coefficient estimated by this model to the soil moisture.

Leconte *et al.* [61] applied the Dubois model to a series of RADARSAT-1 images acquired over an agricultural area. A soil surface roughness map was first retrieved from a SAR image by inverting the model with known soil moisture. The resulting map was then used to retrieve soil moisture for the remaining SAR images. Good agreement was observed between watershed-scale soil moisture values and measurements averaged for all sampled fields; however, considerable scatter was found between observed and SAR-derived soil moisture estimates at the field scale. Like Leconte *et al.* [61], many studies have reported better soil moisture retrieval at the watershed scale than at the field scale. Thoma *et al.* [98] developed an operational approach to determine the optimal spatial resolution for the required application accuracy and found that the optimum ground resolution will depend on the spatial distribution of land surface features that affect radar backscatter. This statistical approach has high potential for application because it does not rely on ground verification of soil moisture for validation but only requires a satellite image and average roughness parameters of the site.

### 2.3. Physically-based models

A number of theoretically rigorous and approximate solutions for electromagnetic scattering from rough surfaces, described as stochastic random processes, have been developed over the past decades (for a topical review see [99,100]). Several of these backscatter models have failed because of the difficulty in describing the soil roughness [101], leading to many studies investigating the problem of defining optimal parameters that describe surface roughness [19-21,102,103].

The most popular approximate scattering models are the Small Perturbation Model [104], Kirchhoff Approximations [105], the Small Slope Approximation (SSA) model [106,107], and the Integral Equation Model (IEM) [15,40] (and its amended version the Advanced Integral Equation Model (AIEM) [108]). Amongst those with a wider validity range in terms of roughness parameters are the SSA and IEM/AIEM

The Integral Equation Model encompasses the Kirchhoff and small perturbation models in the high and low frequency regions respectively [15,28,40,97]. It is thus able to address a wide range of bare soil roughness states, with an expression that is simple to calculate. IEM calculates the backscattering coefficient *σ*^0^ of a bare soil, given the radar properties (wavelength, polarization), surface characteristics (dielectric constant and surface roughness) and local incidence angle. The theoretical derivation of the IEM starts from the Stratton-Chu integral which describes the scattered electric field *E_s_* observed at the sensor in terms of the tangential electric and magnetic fields at the soil surface. Because the Stratton-Chu integral is complex some approximations as described in Fung [15] have to be made in order to arrive at an analytical solution. As noted by Hsieh *et al.* [109], the validity of these simplifying assumptions has to be justified by comparisons with measurements from statistically known surfaces. IEM also neglects scattering from the sub-surface soil volume which may be important for dry soil conditions and long wavelengths [110].

In order to invert the IEM to dielectric constants (or soil moisture if a model that describes the dielectric constant-soil moisture relation is applied, e.g. [23,24]), several algorithms have been developed, based on the fitting of IEM numerical simulations for a wide range of roughness and soil moisture conditions, including Look-Up Tables (LUT) (e.g. [41,48]), neural networks (e.g. [56]), or the method of least squares (e.g. [29,46,91]).

For bare soil studies, the IEM has become the most widely used scattering model [6]. The validity range of the single scattering approximation of the IEM was defined as *ks* < 3 [40], however, Baghdadi and Zribi [94] found that the model was also applicable outside the presumed validity range.

The IEM has been validated successfully at fine scales in a laboratory setting [109,111-113]. However, for real world applications, contradicting results have been reported [17,36,59,65,94,114]. The reason therefore can be found in the fact that agricultural soils show a large intra-field variability of roughness and moisture conditions that are usually not accounted for in direct scattering models. Zribi and Dechambre [52] compared IEM simulations with data acquired during several campaigns and found a limited applicability of the model. Baghdadi and Zribi [94] observed that IEM frequently overestimated the backscattering in HH polarization, except for data that corresponded to soil moisture contents larger than 35%, where the simulated data showed strong fluctuations. At C-band, the errors observed appeared to be of the same order of magnitude for all surface roughness RMS values between 0.5 and 5 cm, all soil moisture contents between 5 and 35% and all incidence angles ranging between 20° and 48° [94]. For VV polarization, only small overestimations were observed, and largest errors were found for wet soils (*Mv* > 30%) and large incidence angles (*θ*> 44°).

Rakotoarivony *et al.* [17] and Zribi *et al.* [65] observed a better performance of the IEM for simulating C-band HH polarized backscattering over smooth areas than over rough areas. They found an overestimation of about 2 dB over smooth surfaces, which slightly increased with incidence angle. However, contradictory results were found (*i.e.* an underestimation of the HH backscattering coefficient) by Baghdadi and Zribi [94]. Mattia *et al.* [36] showed that IEM simulations tend to overestimate the backscattering coefficient of smooth fields by about 3 dB in C- and L-bands. Baghdadi *et al.* [63] found that IEM results were not accurate, irrespective of the ACF used.

According to Zribi and Dechambre [52] the difficulties in simulating the radar backscattering coefficient as observed for natural surfaces can be attributed to two factors: first, the physical approximations introduced in the model are not *a posteriori* verified [15] and second, the mathematical description of natural surfaces is still insufficient. Since surface roughness from agricultural fields may show multi-scale effects, which were not accounted for in the original IEM concept, Mattia and Le Toan [22] reformulated the IEM such that scattering from a surface roughness described by a multiscale fractal random process could be simulated. They therefore no longer use the classical roughness parameters *s* and *l*, but introduced a new set of parameters that were related to multi-scale surface properties. One result obtained with this novel roughness description was that the backscattering from very smooth agricultural soils could be predicted better [115].

Other improvements of the IEM have been reported: Boisvert *et al.* [57] and Weimann [116] adapted the IEM for penetration depth, Bindlish and Barros [117] included a vegetation scattering component; Shi *et al.* [118] introduced a new class of ACF; Wu *et al.* [119] introduced a transition model for the reflection coefficient that was further validated by Fung and Chen [97]; and Chen *et al.* [120] improved the multiple scattering description. Further modifications the IEM were introduced by Wu and Chen [121], which involved new expressions for the single and multiple scattering and a replacement of the Fresnel reflection coefficient by a transition function that takes surface roughness and permittivity into account. However, and notwithstanding the improvements introduced, the majority of retrieval studies still use the original version of IEM [94].

## 3. Soil roughness characterization

Soil roughness can be considered as a stochastic varying height of the soil surface towards a reference surface [4]. This reference surface can be the unperturbed surface of a periodic pattern (e.g. row structures of a tilled soil surface) or can be the mean surface if only random variations exist. In fact, roughness can be considered as the sum of different components corresponding to different scales: (1) individual soil aggregates and grains and (2) soil clods, which represent the random component, and (3) furrows or tillage rows and (4) topographic trends, which constitute the reference surface. For many agricultural fields, the roughness will depend on the direction: ploughed fields will have a different reference surface and roughness in the row direction compared to the direction perpendicular to it. The orientation of the rows towards the flight line of the satellite will also be important. Soares *et al.* [122] show a bimodal distribution of the radar backscattering coefficient for a partly ploughed field. This bimodality is clearly caused by the change in roughness in both parts of the field. Sano *et al.* [123] reported that the orientation of furrows orthogonal versus parallel to the flight line of the satellite provoked an 8-dB difference in the radar backscattering coefficient for fields of similar soil moisture. Yet, it is often assumed that the roughness is isotropic, and therefore, generally, roughness is analyzed in only one direction (along transects) and obtained roughness values are used for the parameterization of the backscattering models.

Generally, the characterization of surface roughness is obtained from the analysis of height variations observed along transects [48], from which, commonly, the RMS height, correlation length and autocorrelation function are calculated as input to most backscatter models [41,101]. Extensive research has been performed with respect to surface roughness characterization and one major problem is the scale dependency of the roughness parameters. The model describing soil roughness should thus have scale dependent characteristics [124-127]. A problem may therefore arise when one wants to use this property in backscatter models. For inversion studies, the easiest choice is to adopt a single-scale roughness description and seek for an equivalent description of the backscattering coefficient in terms of *s* and *l*, where the latter is usually regarded as a fitting parameter [55,128]. As such, we will focus on the commonly used roughness parameters, *s*, *l* and ACF that characterize single-scale roughness.

### 3.1. RMS height

For discrete one-dimensional surface roughness profiles consisting of *N* points with surface height *z_i_*, the RMS height, *s*, is calculated as [4]:

(1) $$s=\sqrt{\frac{1}{N}\left[\left(\sum_{i=1}^{N}z_{i}^{2}\right)-N{\bar{z}}^{2}\right]},$$

where

(2) $$\bar{z}=\frac{1}{N}\sum_{i=1}^{N}z_{i}.$$

In order to obtain a consistent RMS height measurement, Bryant *et al.* [48] concluded that at least 20 transect measurements of 3 meter in length are necessary. They also found that detrending the transects highly influenced the RMS value. Furthermore, generally an increase in RMS height is found for increasing profile lengths [50,103,129]. For single-scale processes, the RMS values increase asymptotically to a constant value for increasing profile lengths, whereas for multi-scale processes, this behavior is not found.

The relationship between RMS height and environmental variables such as tillage and soil texture has been extensively studied in the past [20,51,130,131]. For agricultural areas, the RMS values generally range between 0.25 cm (sown fields) and 4 cm (ploughed fields) [35]. Álvarez-Mozos *et al.* [132] derived with a 1-m pin profilometer average RMS values ranging between 0.47 cm for rolled fields and 2.6 cm for ploughed fields. Zhixiong *et al.* [51] investigated different tillages on a loam soil in the Netherlands using 1-m roughness profiles, and found RMS values of approximately 1.5 cm for harrowed and rolled surfaces, and values ranging between 2.2 and 4.1 cm for ploughed surfaces, which did not deviate much across the studied fields.

### 3.2. Correlation length

The correlation length, *l*, describes the horizontal distance over which the surface profile is autocorrelated with a value larger than 1/*e* (≅ 0.368) [4,133]. Although this definition seems very simple, measurements of the correlation length have been problematic and difficult to interpret [20,49,50,65,134]. The obtained values are highly variable [103] and depend on the length of the transect used [48,103,129], increasing asymptotically to a constant value with increasing profile length.

Various values for an optimum profile length have been suggested for measuring correlation length, varying from a couple of meters [50] to more than 50 m [103]. Yet, since these contrasting lengths lead to different correlation lengths (and RMS heights), it is obvious that consistent roughness values cannot be estimated for the parameterization of IEM [41].

Baghdadi *et al.* [35] mentioned that correlation lengths in agricultural areas generally vary between 2 and 20 cm. Using a 1-m profiler, Álvarez-Mozos *et al.* [132] measured average correlation lengths ranging between 2.44 cm for rolled surfaces to 7.41 cm for ploughed fields. Similar values were reported by Davidson *et al.* [20], who found average correlation lengths, determined from a multi-site database of 1-m profiles, ranged from 3.7 cm for seedbed to 6.9 cm for ploughed surfaces. From this database, they discovered that higher RMS values were generally associated with increasing correlation lengths. Yet, this correlation cannot be predicted by single-scale roughness theory, but should be explained by the differences in soil clod sizes that are associated with different tillages [51].

### 3.3. Autocorrelation function

The normalized autocorrelation function, for lags *ξ* = *j*Δ*x*, and Δ*x* the spatial resolution of the profile, is given by:

(3) $$\rho (\xi )=\frac{\sum_{i=1}^{N-j}z_{i}z_{i+j}}{\sum_{i=1}^{N}z_{i}^{2}}.$$

Alternatively, the ACF can be calculated as the inverse Fourier transformation of the power spectral density [20,21,51].

In order to fully characterize the ACF of a surface, a discretization interval, used to sample the profile, should be at least as small as one tenth of the correlation length [4,135], as the spatial resolution influences the measurement of the surface correlations [133].

**Figure 2.**
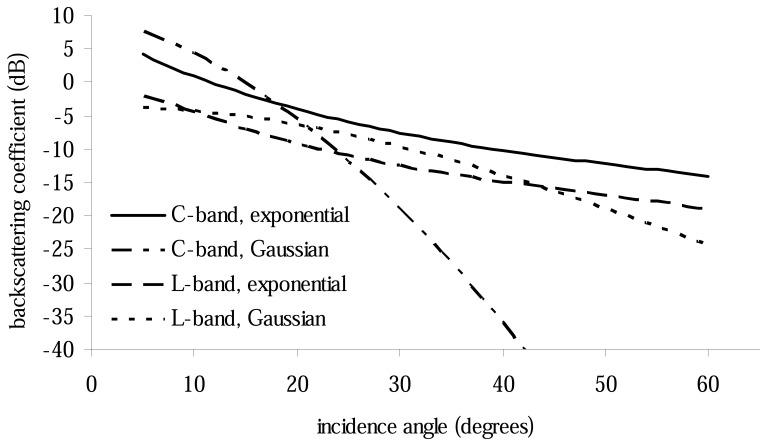
Modeled backscattering coefficient as a function of local incidence angle for C- and L-band VV-configurations and different shapes (exponential and Gaussian) of the autocorrelation function, for a soil having a moisture content of 20 vol%, and roughness parameters (*s*,*l*) = (1 cm,10 cm).

In backscatter models, often two types of ACFs are used [15], i.e. the exponential and the Gaussian autocorrelation function. The exponential ACF is given by:

(4) $$\text{ACF}(\xi )=e^{-\left|\xi \right|/l},$$

and the Gaussian function is defined as:

(5) $$\text{ACF}(\xi )=e^{-\xi^{2}/l^{2}},$$

with *l* the correlation length. Depending on which ACF is chosen, IEM produces strongly different results as demonstrated by Figure 2 which shows the results of the exponential and Gaussian functions for the same setting of *s* and *l*.

Compared to the Gaussian ACF, the exponential one is characterized by smaller correlations at small lags. This causes that exponential ACFs to better describe the micro-roughness in the profile than Gaussian ACFs [133]. However, since these theoretical descriptions do not always describe the roughness of natural surfaces very well, Li *et al.* [136] introduced a generalized power law spectrum that covered both types of ACFs. For agricultural soils, however, different studies found that the ACF was well approximated by exponential correlation functions [19,103,124,129,135,137]; yet, some of the agricultural fields may deviate from the exponential ACF especially at higher lags [51,138]. In case of multi-scale roughness, the ACFs obtained from relatively short profiles showed an exponential decay for small lags [22]. For a single-scale roughness, a better fit between the theoretical and the experimental ACF should be obtained [51].

Following Zhixiong *et al.* [51], the autocorrelation functions can be highly variable and appear to be unrelated to surface roughness conditions for seemingly homogeneous agricultural fields. This can have important implications for the simulation of radar data, or the retrieval of soil moisture, as was demonstrated by Altese *et al.* [114] in a sensitivity analysis.

## 4. Impact of *in situ* roughness characterization on soil moisture retrieval

Although the characterization of soil roughness seems a fairly straightforward methodology, the parameterization faces many problems. One major problem is that roughness parameters often show little or no spatial dependency. In other words, surface height measurements and derived roughness parameters taken at one position often do not, or only poorly, represent their surrounding area which makes them physically meaningless. For example, this was observed by Lehrsch at al. [139] who carried out a semivariogram analysis of eight different roughness indices based on non-contact profiler measurements over 1× 1 m plots. Ogilvy [133] warned that roughness parameters often contain errors due to the measurement process. Mattia *et al.* [21] recognized two main sources of errors that affect roughness measurements: truncation errors, which are caused by recording with relative short profiles, and profiler errors, which are introduced by the intrinsic limitations of the measurement method used. Errors may thus be caused by the choice of profile length, the discretization interval, and the instrument resolution, but also by the overall shape of the profile, the assumptions made with respect to spatial variability and temporal stability. Furthermore, when two-dimensional surface properties are deduced from one-dimensional measurements, the surface properties (such as RMS height) are most likely underestimated as the profiles will generally not record the extrema of the surface [133]. This underestimation will further be enhanced when extracting a mean surface from the measurements, as this mean surface does not necessarily correspond to the intersection of that profile with the mean plane through the two-dimensional surface from which it was taken [133].

In the following sections, a review on the different sources of errors will be given, and when available from literature, the influence of these errors on soil moisture retrieval will be discussed.

### 4.1. Techniques

Several methods have been suggested for estimating the roughness parameters. These can be subdivided in two groups [21]: contact instruments, where there is a physical contact between the instrument and the soil surface and noncontact instruments for which there is no physical contact. Within the first group, meshboard (e.g. [21,140]), and pin profilometer (e.g. [29,41,138,141-145]) can be catalogued.

The meshboard technique involves inserting a gridded board in the soil and making a picture after which it is digitized. The main advantages of a meshboard are its low cost and the fact that it is easy to make. A major disadvantage of the instrument is that it is quite difficult to insert the meshboard sufficiently deep into a rough soil (i.e. the meshboard over the total length needs to be inserted in the soil) without disturbing the roughness, especially, when the soil is compacted. Meshboard measurements are typically affected by parallax errors which are caused by the fact that the picture of the intersection of the soil and the meshboard cannot be taken at ground level, but generally is taken at a height of about 90 cm [21]. Further errors are introduced when processing the images in order to obtain the roughness profile due to geometrical distortions and the on-screen digitalization process [21].

The pin profiler is constructed out of a number of vertically movable pins which are lowered onto the ground surface [146]. The position of the pins, which follow the soil profile, is registered either electronically or is photographed and later digitized [48,147-149]. The main disadvantage of this instrument is the potentially destructive effect of the pins, especially on loose grains or wet soils [146,150], which may influence the correct description of the soil surface. Other disadvantages consist of the imperfect parallelism between needles and the finite dimension of the needle tips [21,151,152]. Furthermore, these instruments have a limited resolution, which is typically about 1-2 mm in vertical direction [150] and 1-2.5 cm in horizontal direction [36,52,150].

Noncontact instruments include laser techniques (e.g. [14,19,153,154]), photogrammetry [137,155-157], acoustic backscatter [158], infrared [159] and ultrasonic techniques [160]. For radar remote sensing studies, the laser profiler is the non-contact technique that is mostly used, and therefore, only this type of instrument will be further discussed.

A laser profiler makes use of a laser beam measuring the distance between a horizontally positioned rail, on which the carriage with the laser beam moves, and the soil surface. The main advantage of this instrument is that it allows for an accurate measurement of the roughness profile having a sufficient horizontal resolution. Yet, these instruments are also characterized by different disadvantages including the interference of light from other sources [125,154], the fact that they are not able to distinguish between changes in topography and changes in optical reflectivity of surfaces, the sensitivity to external disturbances, particularly to wind effects, and the fact that they may suffer from errors introduced by multiple reflections on very rough surfaces or by the presence of green or dry vegetation elements along the path [21]. Laser profilers are characterized by resolutions in the vertical direction from 0.1 to 0.5 mm and in the horizontal direction between 0.1 and 2 mm [150].

Unfortunately, a thorough investigation comparing measurement techniques and accuracy assessment has not been performed yet [48]. In a study on the comparison of meshboard, pin profiler and laser profilometer measurements, Mattia *et al.* [21] found that there is a good agreement, i.e. a relative error less than 8%, between correlation length estimates derived from pin profilers and laser profilers, irrespective of profile length. For surface height variances, the parameters estimated using a pin profiler lead to an overestimation for relatively smooth soils [21]. This finding was not supported by an experiment conducted by Bryant *et al.* [48], where a close one-to-one relationship was found between the RMS height measurements made by both instruments, characterized by a determination coefficient (R^2^) of 0.6. For meshboards, a significant error was found in the roughness measurements (compared to laser profiler measurements), which, according to Mattia *et al.* [21], is most likely to be attributed to errors introduced during the image processing on the digital photos and the subsequent digitization process.

In order to fully comprehend the effect of roughness characterization from different measurement techniques on soil moisture retrieval from SAR, an in-depth study is required that compares the different techniques over the same sites for different roughness situations. Some preliminary results, based on a study that only focused on the RMS height measurements from pin profiler and laser scanning, have been presented by Bryant *et al.* [48]. They concluded that soil moisture predictions suffered errors on the order of several volumetric percentages when using pin profiler measurements instead of laser derived roughness RMS height. They found that the largest differences in soil moisture prediction varied with sensor configuration, surface condition and measurement protocols and generally exceeded 20% of soil moisture prediction.

### 4.2. Preprocessing

The soil roughness parameters describe the statistical variation of the stochastic varying surface height towards a reference surface. Generally, only the first order component from the measured profile is removed. This assumes that the reference surface is a plane, which is not necessarily horizontal, and accounts for the fact that the measurement device may have been slightly tilted with respect to the reference surface. This assumption is only valid when short profiles are measured [103], but for longer profiles, it can be necessary to consider a curved surface, and account for the shape of reference surface in the backscattering model [4]. Alternatively, a multi-scale roughness model can be assumed as, according to Davidson *et al.* [19], the topography-related component in the roughness profile introduces a multi-scale fractal process, while the tillage induced roughness deviations with respect to the curved reference surface are described by a single-scale process [103].

In order to parameterize these single-scale roughness deviations, one should filter out the curved reference surface. Different methodologies can be applied such as detrending using piecewise linear regressions, applying a highpass filter, applying a moving average filter, and detrending using a higher order polynomial [103]. Generally, only the overall first order trend is removed from the observed profiles (e.g. [20]). For longer profiles, such technique may not be sufficient as the roughness parameters keep increasing with increasing transect length (revealing a multi-scale effect). Bryant *et al.* [48] found that removing the linear component of 1-m subprofiles allowed for obtaining relatively stable RMS height values. Callens *et al.* [103] did not select this technique as it may introduce incorrect jumps between the selected subprofiles, but used a third-order polynomial detrending to remove topographical effects observed in 25-m long roughness profiles.

Bryant *et al.* [48] demonstrated that the impact of detrending technique on the retrieval of soil moisture may be extremely large. They reported differences in retrieval of more than 9 vol%, indicating that accurately removing underlying topographic effects is of major importance for soil moisture retrieval studies that depend on soil roughness parameters.

### 4.3. Measurement accuracy

Bryant et al [48] stated that the accuracy of the measurements is in many cases the limiting factor in the accuracy of the soil moisture predictions. The accuracy of the measurements can be subdivided in: 1) horizontal resolution, 2) vertical resolution, and 3) human-based error during digitization.

#### 4.3.1. Horizontal resolution

The horizontal resolution is defined by the instrument that is used. For laser profilers, the horizontal distance between two measurements generally ranges between 1 mm [150] and 5 mm [19], whereas for pin profilers, horizontal resolutions of 2 mm [150] up to 2 cm [63] have been reported. Ogilvy and Foster [135] showed that subsampling in the horizontal direction mainly causes a change in slope of the ACF around zero. Coarser resolution instruments cause that the roughness profile is sampled at an insufficient rate and therefore, very small structures are not represented in the obtained roughness profile. This may result in an underestimation of the high frequency component, which can introduce a different shape of the ACF around the origin. Mattia *et al.* [21] state that this should not have a significant impact on the correct estimate of the correlation length. Yet, for lags smaller than the correlation length, both autocorrelation functions differently describe the surface, where the Gaussian ACF describes a smoother surface (less micro-variability) than the exponential one, even with the same correlation length. The latter may result in different sensitivities of the backscattered signal to soil roughness [114].

In order to prevent major errors in the estimations of roughness parameters, Oh and Hong [138] suggested using a sampling distance smaller than 0.2 times the correlation length in order to obtain a precision of ±5% for RMS height and correlation length. Yet, Ogilvy [133] and Ulaby *et al.* [4] advised to use a horizontal spacing equal to one tenth of the correlation length, such that the total ACF would be characterized accurately. The impact of errors introduced in the roughness parameters on the soil moisture retrieval has, to the knowledge of the authors, not been investigated yet.

#### 4.3.2. Vertical resolution

Although the vertical resolution is an instrument property introducing errors in the measured profile, little attention has been given to the effect of this accuracy on the determination of roughness parameters or the impact on the soil moisture retrieval. Values with respect to the vertical accuracy of different instruments are rarely published. Jester and Klik [150] mentioned that vertical resolutions vary from less than 1 mm for non-contact measurement techniques, such as laser profilometers, to 2.5 mm for instruments that require a contact with the surface. Vertical resolutions have been reported as large as 0.5 cm for needle profilers [36] and 0.3 cm for laser scanners [48]. However, the propagation of this resolution error in roughness parameters and inverted soil moisture values has, to the authors' knowledge, not been assessed.

#### 4.3.3. Digitization error

Both the non-electronic pin profiler and the meshboard technique require a digitization. With pin profilers, the position of the upper part of the pins is photographed and digitized, whereas, the soil surface is immediately digitized when using a meshboard. Archer and Wadge [161] have shown that the influence of the digitization of pin profiler pictures is negligible on RMS height. Further, the human-induced error in the digitization process can be circumvented by electronically processing the photographs of the pin profiler to locate the upper part of the pins [48].

Since the digitization of pins is much less subjective then making a difference between soil and plate when using meshboard pictures, D'Haese *et al.* [162] digitized the same profile ten times and found a coefficient of variation of 1.7% on the RMS height and approximately 6.5% on correlation length, for an average (*s*,*l*) of (0.96 cm,10.2 cm). If 12 different people digitized the same profile, similar average roughness values were obtained, i.e. (*s*,*l*) = (0.96 cm,10.6 cm), and a coefficient of variation of 4.52% and 4.51% for respectively RMS height and correlation length was found. Although these examples are statistically not representative, one could conclude that errors introduced in the digitization process are very small, and therefore, although not assessed, the impact on soil moisture retrieval is expected to be small.

### 4.4. Profile length

It is well known that the values of the roughness parameters depend on the profile length used [21,129,163]. Callens *et al.* [103] found that for short profiles, generally a severe underestimation of the roughness parameters can be expected, and therefore, major errors in soil moisture retrieval can be expected when using roughness values obtained from different profile lengths. Nevertheless, Davidson *et al.* [20] obtained a reasonable agreement between ERS data and IEM simulations using roughness parameters measured with 1 m profiles.

Smooth profiles required a minimum length of 10 m to get an estimation of the RMS height that was comparable to what was obtained when 25-m profiles were analyzed, whereas for rough profiles, 5-m profiles seem sufficient. Zhixiong *et al.* [51] reported an overall increase in RMS height by a factor of 1.2 to 1.4 when comparing data obtained from 5-m profiles with those derived from 0.5-m profiles. Based on a theoretical study, Oh and Kay [129] deduced that profiles of at least 40 times the correlation length are needed in order to obtain the RMS height with a precision of ±10% of their mean value.

In order to get accurate estimations of the correlation length, much longer profiles are necessary. Furthermore, the increase rate for the correlation length with increasing profile lengths is larger than for the RMS height [138]. For short profiles, Callens *et al.* [103] found significant underestimations of the correlation length, when compared to the asymptotic value corresponding to large profile lengths. According to Oh and Kay [129], profile lengths of at least 200 times the correlation length are required to get estimations of correlation length with a precision of ±10%.

Sayles and Thomas [164] suggested that the surface roughness variance (*s*^2^) is proportional to the profile length. Baghdadi *et al.* [50] discovered a relationship of the form:

(6) $$s=\gamma (1-e^{-\eta L}),$$

with *η* and *γ* two calibration constants and *γ* corresponds to the asymptotical RMS-value (obtained for profile lengths *L* → ∞). Callens *et al.* [103] further relaxed this equation, and approximated the relationship between *s* and *L* following:

(7) $$s=a-be^{-\eta L},$$

where *a* and *b* are parameters obtained through a least squares fitting. They furthermore suggested a similar relationship for describing the dependency of the correlation length on *L*. They used both relationships to estimate the asymptotical value of both roughness parameters from 4-m profiles, such that infeasible long profile lengths (extending 25 m in some cases) are no longer required.

Despite the different studies devoted to this scaling behavior of the roughness parameters, an assessment of the errors made on the soil moisture retrieval when applying roughness values obtained from different profile lengths has not been reported yet. Such a study would be extremely useful in order to determine the profile lengths needed such that roughness parameters can be estimated at a scale that is relevant for the scattering process.

### 4.5. Number of measurements

Baghdadi *et al.* [35] mentioned that the measurement precision at a certain profile length can be improved by averaging multiple profiles. They found that, for a correlation length varying between 2 and 20 cm, and for 10 averaged profiles, 2-m profiles provided a precision better than ±5% for RMS height and between ±5% and ±15% for correlation length. Using 1-m profiles, the precision after averaging over 10 profiles can reach ±10% for RMS height and ±20% for correlation length. Bryant *et al.* [48] reported that the heterogeneity of natural surfaces required at least 20 profiles of 3 m in length in order to get representative RMS heights.

Ogilvy [163] demonstrated that the variability of these roughness parameters also depends on the profile length. Unless the surface profile exhibits multi-scale roughness, this variability generally decreases with increasing profile lengths [19,103,129]. This decrease in variability implies that fewer profiles are needed to get an accurate estimate of the roughness parameters [103], in case of longer profiles.

The effect of averaging soil roughness parameters from a different number of profiles on the soil moisture retrieval was assessed by Bryant *et al.* [48]. They discovered differences in soil moisture ranging up to 3.6 vol% when comparing retrievals obtained with roughness parameters determined from 10 and 20 profiles. Due to the increasing sensitivity of the backscattering coefficient to roughness, their results depended on the incidence angle used (larger incidence angles caused larger errors).

### 4.6. Spatial variability of soil roughness

Álvarez-Mozos *et al.* [165] studied the spatial variability of roughness at the field scale and its impact on soil moisture retrieval for different fields within a Spanish catchment. Depending on the tillage state, coefficients of variation for field-average RMS height and correlation length of respectively 16 to 25% and 38 to 94% were obtained. This large in-field variability of roughness parameters demonstrated that errors in IEM calculated backscatter values could easily be higher than 2 dB, especially for incidence angles above 30° and L-band data, as well as for C-band data with incidence angles below 15° or above 45°. Those errors were translated into inaccuracies in retrieved soil moisture that could easily reach 10 vol% especially for wet soils, due to the lower sensitivity of the backscattering coefficient to soil moisture for wet conditions. Based on these results, it is obvious that accurate measurements of roughness parameters are required, along with their in-field variability, in order to retrieve useful soil moisture estimates [165].

### 4.7. Temporal changes of soil roughness

Different studies (e.g. [132,166,167]) assume that soil roughness remains more or less constant in time (unless a tillage is performed), such that the same roughness parameters can be used for inversion during a longer period. However, this assumption may not be valid, since incident rainfall may cause changes in soil roughness [131,150,168]. Onstad and Zobeck [131] proposed a simple empirical model which expresses that soil roughness (linked to RMS height) decays exponentially with increasing amount of rainfall, or cumulative kinetic energy contained in the rainfall. Zhixiong *et al.* [51] mentioned that this relationship is further influenced by soil texture and aggregate stability.

During three months, Callens *et al.* [103] measured roughness using 25-m profiles on the same field, on which different tillages were applied and found that not all types of tillages showed the exponential decay in RMS height. This was attributed to the fact that the tillage was performed a month before the measurements took place. During this period, the major roughness changes due to splash erosion had already occurred, resulting in roughness which could be considered as temporally constant. For the correlation length, one would expect an increase in time, as the soil erosion smoothens the surface, however, Callens *et al.* [103] found no consistent temporal trends.

Álvarez-Mozos *et al.* [165] also investigated the temporal dynamics of soil roughness on seedbed fields. They found that generally, *s* decreased whereas an increase in correlation length was observed with time. Even if these temporal changes in roughness were not strongly significant if the in-field spatial variability was taken into account, their influence on the backscattering coefficient can be important because both effects (*s* reductions and *1* increments) contribute to a more specular-like behavior of soils. Therefore, backscattering values increase at low incidence angles and decrease at large incidence angles as the surface smoothens. When studying the impact on soil moisture retrieval, Álvarez-Mozos *et al.* [165] demonstrated from IEM simulations that when temporal dynamics in roughness are not considered, soil moisture tends to be underestimated with values higher than 10 vol%. The errors made are largest for wetter soils and higher incidence angles, due to the fact that for both cases, the signal is less sensitive to soil moisture and more sensitive to roughness.

## 5. Alternative approaches to the roughness problem

### 5.1. Multi-scale processes

There is a general consensus that land surface is shaped by phenomena behaving at many (spatial and/or temporal) scales rather than at a single fundamental scale. For instance, over large scales land surface roughness depends on climatic conditions [169]. Whereas, at medium spatial scales, geophysical processes like erosion [170], runoff [171], weathering [127] or volcanic eruptions [172] have an impact on soil roughness. Furthermore, the large and medium scale phenomena are modulated by anthropogenic small-scale phenomena such as agricultural practices or crop calendar [159], etc. At the other extreme, i.e. at microscale (less than 10 cm), surface microtopography generally depends on soil grain and clod size [125]. As a mathematical framework to represent multi-scale phenomena, self affine random processes (i.e. random fractals) have been exploited (for a systematic treatment see for instance [173,174]). The most well known example of random fractal process is the Gaussian fractional Brownian motion (fBm) process characterized by stationary increments [175,176]. Random fractals are statistically invariant to spatial scale transformations and possess a 1*/f* power spectrum (i.e *S(f) ∝* 1*/f^ν^* where *ν* = (7 − 2*D*) and *D* is the surface fractal dimension). For these random processes, traditional roughness parameters, namely the profile height rms (*s*) and profile correlation length (*l*) are not intrinsic properties of the surface, but depend on the measured profile length [177]. This property can be employed to simply identify multi-scale roughness profiles, provided the profiles are sufficiently long (see for instance [19,21,178,179]). It is worth noting that *1/f* surfaces always possess more important high frequency components than single-scale Gaussian correlated surfaces. On the contrary, they may possess larger or smaller high frequency components than single-scale exponentially correlated surfaces, depending on *ν* (more details can be found in [180,181]).

Based on the aforementioned description, different researchers have tried to describe soil surfaces using band-limited fractals (e.g. [22,53,182-184]). Although the latter helps in better understanding the backscattering process, its application involves measurements of more complicated roughness parameters, causing difficulties for inverting the radar signal [52,54].

### 5.2. Calibration of parameters

Since the correlation lengths assessed from field measurements are generally inaccurate, Baghdadi *et al.* [62] proposed to use an empirical relationship between RMS height and correlation length:

(8) $$l=e^{\alpha s+\beta },$$

where *a* and *β* are calibration constants depending on incidence angle and polarization. This relation was parameterized by fitting the IEM results to radar measurements until a good agreement was obtained. Their results revealed that the calibrated correlation length was strongly related to the RMS height, but that these relations were dependent on the radar configuration. Baghdadi *et al.* [63] found that this relationship was no longer valid when rougher soils were included in the analysis. For an RMS height ranging between 0.25 cm and 5.5 cm, a better agreement between IEM backscatter predictions and observed data was obtained when a power-type relationship of correlation length with RMS height was applied to exponential and fractal autocorrelation functions, expressed as:

(9) $$l=\alpha s^{\beta }.$$

For surfaces characterized by a Gaussian autocorrelation function, they found that a linear relationship better fitted the IEM results:

(10) l=αs+β

In a later study, Baghdadi *et al.* [46] found that the values of *α* and *β* were dependent on the polarization and incidence angle of the SAR observations used, and *α* and *β* values for C-band data were recommended.

Although generally better retrieval results are obtained when using these calibration approaches, this technique does not allow to extrapolate the obtained models, unless the sites on which it is applied are similar to the one used for its development. Rahman *et al.* [41] used the theoretical framework of IEM to determine the relation between RMS height and correlation length and then based the model calibration on a SAR image acquired with dry soil conditions. This eliminated the reliance on *in situ* measurements of surface soil moisture and accounted for the very complex relation between *s* and *l* found in heterogeneous landscapes.

### 5.3. Two-dimensional surface roughness characterization

In the past, soil surface height measurements were almost exclusively taken along one-dimensional profiles. Thus it was not possible to carry out an analysis of the two-dimensional soil surface height field, which may possibly lead to different roughness characterizations then compared to the one-dimensional case. Two-dimensional height maps *z*(*x*, *y*) can be obtained by stereo-image matching or by using (terrestrial) laser scanners. Jester and Klik [150] found that the laser scanner was able to reproduce small aggregates as well as voids in between them, while the digital height model from the stereophotos was smoothed between major aggregates. Unfortunately, these techniques have not yet been applied to study roughness effects on SAR measurements. A first study has only been recently initiated by Perez-Gutierrez *et al.* [186].

The terrestrial measurements may be complemented by airborne laser scanner measurements, which have become the main data source for high quality digital terrain models [187]. Even though airborne laser scanners have laser footprint sizes on the order of 20-100 cm and ranging accuracies of several centimeters, Davenport *et al.* [188,189] showed that it is possible to distinguish different soil treatments by calculating the RMS height after detrending to remove topographic slopes. Novel airborne laser scanners are also capable of recording the complete echo waveform which allows determining the backscatter cross section at the laser frequency [190], which is known to be also very sensitive to soil roughness. Nevertheless, more research is needed before firm conclusions can be drawn.

### 5.4. Multi-image approach

Another approach to overcome roughness parameterization effects in the soil moisture retrieval consists of combining two or more SAR images of different incidence angle with the IEM to separate the effects of soil moisture and roughness for several tillage types [68,191-193]. Based on a theoretical analysis, Fung *et al.* [194] reported that angular SAR measurements could be used to determine roughness parameters for IEM, and furthermore that this approach was preferable to direct ground measurements as this accounts for scale, heterogeneity and resolution.

Zribi and Dechambre [52] found that the difference in backscatter (Δ*σ*^0^) generated by the IEM model with two different incidence angles, keeping all other parameters constant, was proportional to roughness only, expressed as a ratio of *s*^2^/*l* termed the Z-index. Rahman *et al.* [41] showed that it was possible to derive *s* and *l* separately from the Z-index using the IEM with a SAR image acquired with dry soil conditions. The resulting maps of distributed roughness can be used to parameterize IEM for producing surface soil moisture maps without the need for ancillary data [68]. The approach required the use of a SAR image acquired with dry soil conditions and two SAR images from two different view angles. During the time span of the two multi-angular image acquisitions, the surface soil moisture should remain nominally constant. This is an obvious constraint with currently orbiting radar systems which cannot acquire multi-angular imagery during a single overpass. On the other hand, it may be possible to replace two-angle imagery with two-polarization imagery (which can be obtained in a single overpass) in the Zribi-Dechambre formulization.

### 5.5. Using polarimetric data

An alternative way to address the roughness problem is to make use of polarimetric parameters such as, for example, the entropy, the α angle and the anisotropy [44]. This should allow for mapping two soil surface characteristics simultaneously [35]. The underlying idea is that the polarimetric signature of low depolarizing targets (e.g. bare or sparse vegetated fields) is expected to be very sensitive to geometrical properties of the soil surfaces. Then, the polarimetric information can be exploited to maximize the radar sensitivity, for instance, to surface roughness. As a result, a polarimetric feature extremely sensitive to roughness and almost insensitive to moisture is obtained. Mattia *et al.* [36] found that the copolarized correlation coefficient, expressed in a circular polarization basis showed a strong dependence on the roughness state and less to soil moisture content. Schuler *et al.* [195] discovered that the real part of the circular coherence is more sensitive for surface roughness than circular coherence itself. Although further research, mainly limited to the assessment of the roughness parameterization accuracy, is being conducted on this topic, its applicability to current space-borne sensors is limited since the majority of the available sensors are not fully polarimetric.

### 5.6. Using prior knowledge on roughness state

Satalino *et al.* [56] suggest that it would be sufficient to have prior information on the surface roughness about a given area such that the inversion algorithm can be tuned to a specified roughness range, which could be addressed in a fuzzy way through possibility distributions or membership functions [66]. This prior information on surface roughness can be obtained through knowledge of crop calendars from which the tillage state can be deduced [66,130].

Knowledge on the tillage state of a field, however, does not allow for accurate roughness parameterization. On the contrary, there exists a range of roughness values possible for the specific tillage state, and this vague information should be used in the retrieval process in order to determine a range of possible soil moisture values and/or a most likely soil moisture value. Satalino *et al.* [56] trained a neural network such that the backscattering coefficient could be inverted to soil moisture for a given roughness state. Verhoest *et al.* [66] applied the extension principle of Zadeh [196-198] to propagate the uncertainty in the roughness parameters of a certain tillage class through an inverse backscatter model in order to obtain the possibility distribution of soil moisture. The latter function was then used for estimating the soil moisture content and an uncertainty upon its value. Based on the same technique, Verhoest *et al.* [199] developed a fuzzy model that retrieved soil moisture and estimated its uncertainty given a possibilistic roughness description.

The results of Satalino *et al.* [56] and Verhoest *et al.* [66] show that, although uncertainty is allowed in the roughness parameters, good retrieval results can be obtained with accuracies less than 5 vol% at C-band. Of course, this accuracy depends on the uncertainty allowed for in the roughness parameters, and the radar configuration used.

## 6. Conclusions

From an extensive literature study, it is clear that roughness parameterization is an important yet problematic issue in SAR-based soil moisture retrieval. Basically, the way roughness needs to be described and measured for the modeling of backscattering is not fully understood, and generally, the problem is simplified through assumptions of single-scale, isotropic roughness.

In most backscatter models, two roughness parameters are required, i.e. the RMS height and the correlation length, and in theoretical models such as the Integral Equation Model (IEM), the shape of the autocorrelation function (ACF) also needs to be known. For natural surfaces, an exponentially decaying function appears to be a reasonable approximation of the ACF, but given the high sensitivity of theoretical models to the selected ACF, even small deviations can cause differences in the calculated backscatter on the order of several decibels. Yet, the largest problems for the parameterization of the roughness are encountered with respect to the correlation length. This parameter is characterized by a very high variability, causing average values of generally a small number of roughness measurements to be characterized by a high uncertainty. Since the RMS height values are less variable between roughness profiles, its parameterization is less problematic.

The profile length used in the field for characterizing roughness highly determines the roughness parameters. Longer profiles result in higher values and a smaller variability for both RMS height and correlation length. Since different parameters may thus result for the same roughness, the profile length used will have an important impact on the retrieval of soil moisture, given the high sensitivity of the backscattered signal to roughness. Therefore, several authors argue that long profiles should be used for an accurate estimation of the roughness parameters. Also, it may be important to collect height measurements in two dimensions. However, it has never been shown at what scale or dimension surface roughness needs to be measured in order to be relevant for an accurate description of the scattering phenomenon. Further research on this issue is thus very important such that adequate roughness parameters can be determined in the field in order to support backscatter modeling or soil moisture retrieval. If not, techniques which try to circumvent the in-field measurement of roughness (because of the uncertainty of its values) will have to be developed in order to make further progress in SAR soil moisture retrieval, like those discussed in Section 5.

If SAR is to be used on an operational scale for soil moisture mapping, then soil roughness parameterization through *in situ* measurements is not an option. New techniques that allow for moisture retrieval need to be further explored that circumvent roughness characterization (e.g., through relating backscatter differences to changes in moisture content) or that allow for roughness characterization based on remote sensing (e.g. polarimetry, multi-angle viewing, laser scanning). Alternatively, ranges of (calibrated) roughness values can be assigned to different tillage practices which can then be used as *a priori* information as input to soil moisture retrieval algorithms.

The advent of new high resolution sensors observing in X- and L-band (TerraSAR-X, CosmoSkyMed and ALOS), and C-band sensors yielding polarimetric data (RADARSAT-2) should allow for a better characterization of surface parameters. It is expected that a combined use of data from these different platforms, can lead to retrieving soil roughness information at a spatial scale relevant to the observed scattering. These roughness values can then be used for operational soil moisture retrieval from SAR imagery.

Several studies have been devoted to improving the roughness characterization, to assessing errors and to estimating scaling behavior of the roughness parameters. However, there is still no comprehensive assessment of the impact of these roughness problems on the soil moisture retrieval. Nevertheless, an improved insight in the roughness parameterization and its impact on soil moisture retrieval is a prerequisite for making further advances in electromagnetic backscatter modeling and soil moisture retrieval. It is important that the obtained soil moisture products are accompanied by an accuracy measure, as such information is of major importance in order to properly assimilate these spatial soil maps into land surface models (e.g., [200]). However, due to differences in sensitivity of the backscattered signal to the soil moisture content or soil roughness, differences in the retrieval accuracy can be expected [66]. In order to address this issue, additional research on the uncertainty assessment of soil moisture retrieval algorithms is required.
